# IL-4/IL-13 remodeling pathway of COVID-19 lung injury

**DOI:** 10.1038/s41598-020-75659-5

**Published:** 2020-10-29

**Authors:** Caroline Busatta Vaz de Paula, Marina Luise Viola de Azevedo, Seigo Nagashima, Ana Paula Camargo Martins, Mineia Alessandra Scaranello Malaquias, Anna Flavia Ribeiro dos Santos Miggiolaro, Jarbas da Silva Motta Júnior, Gibran Avelino, Leticia Arianne Panini do Carmo, Lucas Baena Carstens, Lucia de Noronha

**Affiliations:** 1 School of Medicine, Postgraduate Program of Health Sciences, Pontifícia Universidade Católica do Paraná-PUCPR, Rua Imaculada Conceição, 1155 - Prado Velho, Curitiba, PR Brazil; 2 Laboratory of Experimental Pathology, School of Medicine, Pontifícia Universidade Católica do Paraná-PUCPR, R. Imaculada Conceição, 1155 - Prado Velho, Curitiba, PR Brazil; 3 Hospital Marcelino Champagnat, School of Medicine, Pontifical Catholic University of Paraná-PUCPR, R. Imaculada Conceição, 1155 - Prado Velho, Curitiba, PR Brazil; 4 School of Medicine, Pontifícia Universidade Católica do Paraná-PUCPR, R. Imaculada Conceição, 1155 - Prado Velho, Curitiba, PR Brazil

**Keywords:** Immunopathogenesis, Prognostic markers

## Abstract

The COVID-19 fatality rate is high when compared to the H1N1pdm09 (pandemic Influenza A virus H1N1 subtype) rate, and although both cause an aggravated inflammatory response, the differences in the mechanisms of both pandemic pneumonias need clarification. Thus, our goal was to analyze tissue expression of interleukins 4, 13, (IL-4, IL-13), transforming growth factor-beta (TGF-β), and the number of M2 macrophages (Sphingosine-1) in patients who died by COVID-19, comparing with cases of severe pneumopathy caused by H1N1pdm09, and a control group without lung injury. Six lung biopsy samples of patients who died of SARS-CoV-2 (COVID-19 group) were used and compared with ten lung samples of adults who died from a severe infection of H1N1pdm09 (H1N1 group) and eleven samples of patients who died from different causes without lung injury (CONTROL group). The expression of IL-4, IL-13, TGF-β, and M2 macrophages score (Sphingosine-1) were identified through immunohistochemistry (IHC). Significantly higher IL-4 tissue expression and Sphingosine-1 in M2 macrophages were observed in the COVID-19 group compared to both the H1N1 and the CONTROL groups. A different mechanism of diffuse alveolar damage (DAD) in SARS-CoV-2 compared to H1N1pdm09 infections were observed. IL-4 expression and lung remodeling are phenomena observed in both SARS-CoV-2 and H1N1pdm09. However, SARS-CoV-2 seems to promote lung damage through different mechanisms, such as the scarce participation Th1/Th17 response and the higher participation of the Th2. Understanding and managing the aggravated and ineffective immune response elicited by SARS-CoV-2 merits further clarification to improve treatments propose.

## Introduction

In June 2009, the world was facing the first pandemic disease of the twenty-first century caused by a respiratory virus, the pandemic Influenza A virus H1N1 subtype (H1N1pdm09). In February 2010, the number of cases estimated by the Center for Disease Control and Prevention (CDC) was around 59 million, with approximately 12 thousand deaths^1^. Eleven years later, the world is faced again with a pandemic disease called COVID-19 once more caused by another respiratory virus, the SARS-CoV-2 new coronavirus^2^. Until June 15 of 2020, the fatality rate would be 5.4%^3^. In comparison, the mortality rate of H1N1pdm09 infection was less than 1%^4^.

Critical state COVID-19 patients require the use of mechanical ventilation^5^, and a study reported that about 9 to 11% of hospitalized patients, mainly elderly, required mechanical ventilation due to complications^6^. Severe conditions can lead to acute respiratory distress syndrome (ARDS)^7^ with diffuse alveolar damage (DAD) characterized interstitial septal edema and hyaline membrane in the acute phase and the proliferation of fibroblasts and septal fibrosis in the chronic remodeling phase^8^.

The inflammatory cytokine storm described in COVID-19 appears to be closely related to the development and progression of ARDS. The massive cell destruction caused by SARS-CoV-2 would exacerbate cytokines releasing due to the activation of macrophages and a delay in the recruitment of TCD8^+^ lymphocytes, triggering an inadequate Th1 response^9–11^.

The DAD fibrotic phase is understood as a repair mechanism induced by the activation of M2 macrophages triggered by Th2 response and TCD4^+^ lymphocytes. Th2 cell's functions are mediated by interleukin 4 (IL-4), which activates interleukin-13 (IL-13). Both interleukins differentiate M2 macrophages with consequent fibrosis and release growth factors, such as the transforming growth factor-beta (TGF-β)^4,12^.

In the absence of proven effective treatment for COVID-19, current therapy consists of supportive care. Besides, many patients have received off-label medications, including antiretrovirals, antiparasitic, anti-inflammatory drugs, and convalescent plasma^13^.

In this paper, post-mortem biopsies of COVID-19 patients were compared to patients who died of H1N1pdm09 and the control group to understand the role of IL-14, IL-13, TGF-β, and M2 macrophages recruitment in the cytokine storm and DAD pathogenesis in order to analyze the remodeling phase and its comorbidities.

Therefore, we sought to evaluate the histopathological and physiological differences between SARS-CoV-2 and H1N1pdm09, observing cytokine's expression to understand the mechanism leading to COVID-19 from mild to lethal disease as a result of immune dysfunction, thus being a requirement to identify possible treatments for critical disease.

## Methods

### Ethical approvals

The presented study was approved by the National Research Ethics Committee (Conselho Nacional de Ética em Pesquisa—CONEP), protocol number 3.944.734/2020, and 2.550.445/2018. The authors confirm that all methods were carried out following relevant guidelines and regulations.

Families permitted the post-mortem biopsy of the cases of COVID-19, H1N1pdm09, and CONTROL groups; and signed the informed consent forms.

Samples were not obtained from prisoners, and the sample collection followed all relevant ethics and safety protocols.

### Samples

Clinical data were obtained from medical records during hospitalization in the Intensive Care Unit (ICU) at the Hospital Marcelino Champagnat in Curitiba, Brazil (n = 6). Testing for COVID-19 was performed on nasopharyngeal swabs taken during ICU hospitalization, and the performed Real-Time Polymerase Chain Reaction (RT-qPCR). The viral genome's amplification was performed with the Invitrogen SuperScript™III Platinum^®^ One-Step qRT-PCR Kit (Catalog number: 11732020, Waltham, MA), were positive for SARS-CoV-2.

The pandemic H1N1 group consists of lung samples from patients whose cause of death was H1N1pdm09 severe acute respiratory infection (n = 10) during the 2009 pandemic. The patients were tested through the fresh samples of lung post-mortem biopsies, and the performed qRT-PCR (a similar technique to that of the COVID-19 group) was positive for H1N1pdm09.

The CONTROL group (n = 11) was composed of necropsy lung samples of patients who died due to cardiovascular and neoplastic disease, not involving lung lesions. The age of the CONTROL group ranged from 18 to 60 years, with mainly male patients, similarly to the pandemic H1N1 and COVID-19 groups.

A minimally invasive lung post-mortem biopsy was performed through a left anterior mini-thoracotomy with upper left lobe lingular segment resection. The resected pieces were 3 × 3 cm.

### Histological and immunohistochemistry analysis

The lung samples provided by post-mortem biopsy were formalin-fixed paraffin-embedded (FFPE) and stained with hematoxylin and eosin—H&E (Harris Hematoxylin: NewProv, Cod. PA203, Pinhais, BR; Eosin: BIOTEC Reagentes Analíticos, Cod. 4371, Pinhais, BR). The immunohistochemistry technique was used to identify the expression of the IL-4, IL-13, TGF-β, and Sphingosine-1 for M2 macrophages scoring (Table 1).

**Table 1:** Resource table.

Antibody	Type	Clone/code	Dilution	Source	Species reactivity	RRID
Anti-Sphingosine-1	Polyclonal/Rabbit	Ab71700	1:200	Abcam	Human, Rat, Mouse^a^	AB_1270891
Anti-IL-4	Polyclonal/Rabbit	PA5-25165	1:200	Thermo Fisher Scientific	Human, Mouse^b^	AB_2542665
Anti-IL-13	Polyclonal/Rabbit	P130-E	1:600	Thermo Fisher Scientific	Human^c^	AB_223471
Anti-TGF-β	Polyclonal/Rabbit	E11262	1:200	Spring	Human^d^	–^e^

^a^https://www.abcam.com/sphk1-antibody-ab71700.html.

^b^https://www.thermofisher.com/antibody/product/PA5-25165.html?CID=AFLAGPA525165.

^c^https://www.thermofisher.com/antibody/product/P130E.html?CID=AFLAG-P130E.

^d^https://www.imtec.be/files/images/Spring%20Bio%20catalogus%20-%20imtec.pdf.

^e^There is not a RRID once the manufacturer (Spring) were merged to another company (Abcam).

The replication of the experiment does not apply to the immunohistochemistry technique. The result is confirmed by the positivity of positive control, a sample known to be positive for a specific antibody allocated together with the patient's samples (lung sample with respiratory syncytial virus (RSV) pneumonia, skin sample with dermatosis, and hyperplastic lymph node).

The IL-4, IL-13, and TGF-beta slides were scanned with Axio Scan.Z1 Scanner (ZEISS, Jena, Germany), and then ZEN Blue Edition (ZEISS, Jena, Germany) utilized to randomly generate the ten high-power fields (HPF = 40X objective). The analysis was blind once the images were randomly generated by the software, with no investigator's interference. The immunopositivity areas were measured by the Image-Pro Plus software version 4.5 (Media Cybernetics, Rockville, MD). Subsequently, these areas were converted into percentages to enable statistical analysis.

The Sphingosine-1 slides were also used to highlight M2 macrophages in ten HPF. The images were chosen randomly from the septum and lumen alveolar, where the M2 macrophages were scored using the modified Allred score method. The semiquantitative analysis was obtained by summing two scores (proportion and intensity of positivity), ranging from 0 to 8. The proportion score is subdivided according to the percentage of stained cells: score 0—0% stained cells, score 1—< 1%, score 2—1–10%, score 3—11–33%, score 4—34–66% and score 5—> 66%. While the intensity of positivity is evaluated: negative—score 0, weak—score 1, moderate—score 2, and strong—score 3.

### Statistical analysis

The comparison of the quantitative variables of the two groups was performed using the non-parametric Kruskal Wallis test. Values of p < 0.05 indicated statistical significance. The data were analyzed using the IBM SPSS Statistics v.20.0 software. Armonk, NY: IBM Corp.

## Results

Clinical characteristics of the COVID-19 (n = 6), H1N1 (n = 10), and CONTROL (n = 11) groups as gender, age, time from hospitalization to death, comorbidities, histopathological patterns, and the tissue expression of IL-4, IL-13, TGF-B, and M2 macrophages score are listed in Table 2.

**Table 2:** Comparison between COVID-19, H1N1, and CONTROL groups according to clinical findings and pathology features.

Data	COVID-19 (N = 6)	H1N1 (N = 10)	Control (N = 11)
Gender	Male (4) 66.6%	Male (8) 80.0%	Male (8) 72.7%
	Female (2) 33.4%	Female (2) 20.0%	Female (3) 27.3%
	0.55*		0.79**
Age (years)^a^	76.5/80.5 (53–87)	43.5/44 (23–61)	42.3/45 (18–60)
	0.005*		0.003**
Time from hospitalization to death (days)^a^	12.8/10 (2–32)	4.70/1.5 (1–19)	–
Comorbidities (number of cases)	Hypertension (4/6) Dyslipidemia (1/6) Hypothyroidism (1/6) Class II obesity (2/6) Dementia (2/6) Diabetes mellitus (1/6) Chronic kidney disease (2/6) Coronary disease (2/6)	–	–
Mechanical ventilation^a^	9.7/8 (0–21)	4.70/1.5 (1–19)	–
Histological pattern of DAD	Interstitial pneumonitis with scarce septal neutrophils, hyaline membrane, with microthrombosis	Interstitial pneumonitis with high septal neutrophils infiltration, with no microthrombosis	Normal septum
Laboratory test 24 h before death (lymphocytes, mg/dl)^a^	1331.50/1045.50 (628.00–3514.00)	–	–
IL-4 tissue expression^a,b^	8.26/9.37 (0.71–13.39)	0.54/0.41 (0.19–1.12)	2.84/2.26 (0.23–7.41)
	0.003*		0.05**
IL-13 tissue expression^a,b^	0.39/0.28 (0.02–1.34)	2.05/1.60 (0.53–5.19)	0.13/0.02 (0.00–0.76)
	0.007*		0.07**
TGF-β tissue expression^a,b^	3.61/1.55 (0.14–13.53)	3.49/3.12 (0.47–8.88)	3.32/2.18 (0.46–9.92)
	0.51*		0.75**
Score of M2 macrophages (Sphingosine-1)^a,c^	2.33/2.00 (2.00–3.00)	3.10/3.00 (2.00–4.00)	1.18/1.00 (1.00–2.00)
	0.05*		0.001**

^a^Average/median (Min–Max).

^b^Tissue expression in percentage per HPF.

^c^Allread score in 10 HPF. *DAD* diffuse alveolar damage.

*p-values obtained were compared between COVID-19 versus H1N1.

**p-values obtained were compared between COVID-19 and CONTROL group; *p*-values were performed using the non-parametric Mann–Whitney test (p < 0.05).

**Figure 1:**
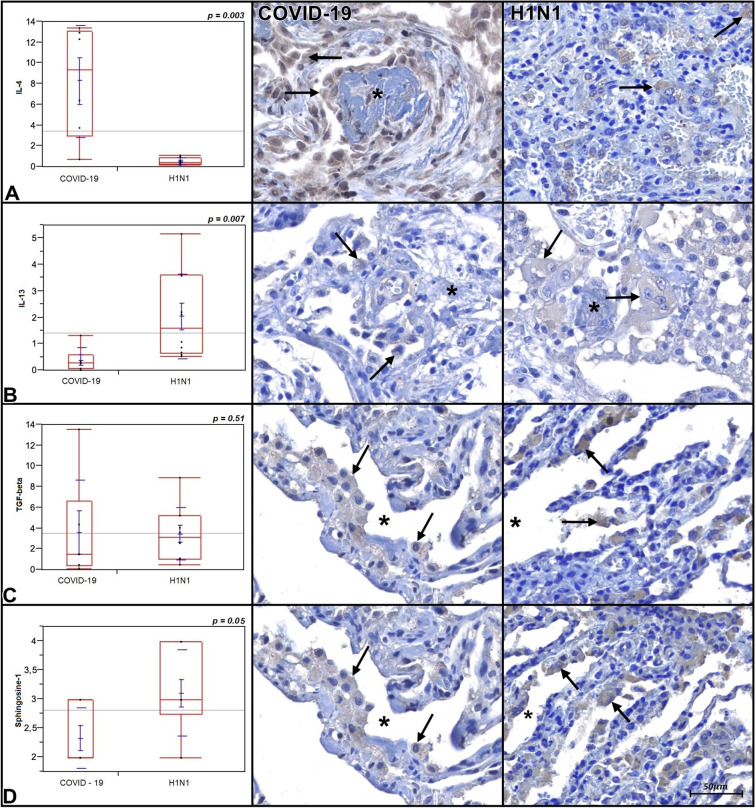
Graphics are showing tissue expression of IL-4, IL-13, TGF-β (percentage per HPF) and M2 macrophages Allred score of COVID-19 and H1N1 groups. Photomicrography is showing alveolar macrophages (arrows) expressing IL-4, IL-13, TGF-β, and Sphingosine-1 (M2 macrophages phenotype) in both groups. Asterisks are showing rests of hyaline membrane forming fibrin plugs (IL-4 and IL-13) and alveolar lumens (TGF-β and Sphingosine-1).

Tissue expression comparison of IL-4, IL-13, TGF-β, and the macrophages (Sphingosine-1) score of the COVID-19 and H1N1 groups are shown in Fig. 1 and Table 1. The COVID-19 group presents statistically significant higher tissue expression of IL-4 compared to H1N1 (*p* = 0.003) and CONTROL groups (*p* = 0.05, borderline). The H1N1 group presents a statistically significant higher tissue expression of IL-13 compared to COVID-19 (*p* = 0.007). No statistically significant differences between COVID-19 and CONTROL groups are shown (*p* = 0.07). The TGF-β tissue expression did not present statistical significance differences when all three groups were tested (*p* = 0.51 and *p* = 0.75, respectively). The M2 macrophages score was statistically significantly higher in the H1N1 group compared to the COVID-19 group (*p* = 0.05). When the COVID-19 group was compared to the CONTROL group, the former shows M2 macrophages score statistically significantly higher (*p* = 0.001).

**Figure 2:**
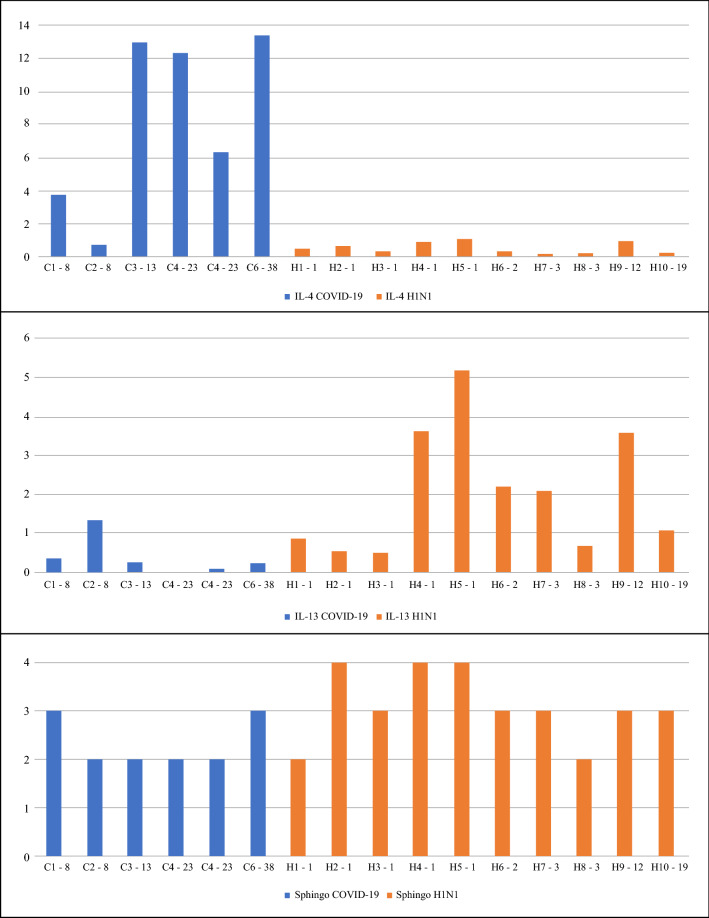
Graphics are showing lung tissue expression of IL-4, IL-13, TGF-β (percentage per HPF) and M2 macrophages Allred score (Sphingosine-1) of COVID-19 and H1N1 groups in a timeline (time from hospitalization to death). *C *COVID19 group following by patient number, *H *H1N1 group following by patient number; the second number refers to the time from hospitalization to death), *Sphingo *Sphingosine-1.

Figure 2 demonstrates the lung tissue expression of IL-4, IL-13, and Sphingosine-1 (M2 macrophages) in all the COVID-19 and H1N1 patients. The IL-4 is consistently higher in COVID-19 patients when compared to H1N1, even if considering different times from hospitalization to death. A contrary result was found for IL-13 and Sphingosine-1.

## Discussion

Regarding the population analyzed, our findings support the literature that shows age as a risk factor and describes comorbidities associated with the severe form of COVID-19 and fatal outcomes^14–16^. Although we have not found a statistical difference in gender distribution, male patients were prevalent (66.6%) in the COVID-19 group (as well as in the H1N1 group = 80%). The COVID-19 and H1N1pdm09 are different pandemic diseases concerning their demographic risk groups, pathophysiology mechanisms, and coinfection prevalence. Contrary to H1N1pdm09, COVID19 lethality is concentrated in older than 65 years of patients-following long periods of mechanical ventilation. Therefore, it is difficult to discard the influence of age, strength and duration of mechanical ventilation in the parameters examined in this report^14–16^. So, it is essential to interpret our findings with caution and validate them in other samples.

Concerning the inflammatory biomarkers studied, the COVID-19 group corroborates the Th2 response predominance, given the higher tissue expression of IL-4 of this group compared to H1N1 and CONTROL groups. The higher score of M2 macrophages in the COVID-19 group compared to the CONTROL group may suggest that the Th2 response is activated. The clinical finding of lymphopenia may support the M2 pathway Th2 response.

Although IL-4 is increased in the COVID-19 group, indicating Th2 response, when assessing IL-13 tissue expression and M2 macrophages score, both were decreased when compared to the H1N1 group (*p* = 0.007 and *p* = 0.05, borderline; respectively), suggesting the Th2 proliferative phase was not triggered (Table 2).

The destruction of epithelial cells in the alveolar space (pneumocytes type I and II) caused by SARS-CoV-2 may lead to macrophages hyperactivation conducting to the cytokines storm. It is suggested that the higher release of interleukin-6 (IL-6) during this initial immune response may suppress T lymphocyte activation, which would explain the presence of lymphopenia in COVID-19 patients. A study with SARS-CoV-2 infected patients in the ICU showed low TCD4^+^ and TCD8^+^ lymphocyte scores and the high IL-6 and TNF-α (tumor necrosis factor-alpha) serological levels. Besides, these patients present high levels of PD-1 (programmed cell death protein 1) that appear to functionally deplete T cells, indicating that the immune system would be tilting abnormally towards Th2 response^9,10,17,18^.

Interleukin-4, the main cytokine of the Th2 immune response, plays a critical role in the Th2 pathway as the effector and inducer of this immune mechanism. Both this interleukin and IL-13 are predominantly associated with fibrogenic inflammatory remodeling, while Th1 cells exert anti-fibrotic activity by secreting gamma interferon (IFN-γ) and interleukin 2 (IL-2)^19^.

Like IL-4, IL-13 actively participates in the Th2 pathway, since both interleukins share the same receptor (IL-4Ra). IL-13 works with IL-4 to induce alternative activation of M2 macrophages (Sphingosine-1), promoting the release of TGF-β and platelet-derived factor. This phase is characterized by the transient expansion of resident fibroblasts and the formation of a temporary matrix and the proliferation of airway progenitor cells and type 2 pneumocytes^9,12,20^.

Although IL-4 and IL-13 are closely linked to the remodeling tissue process, it is suggested that asthmatic patients have a lower risk of developing the severe form of COVID-19 because they have increased levels of both interleukins and promote the suppression of angiotensin-converting enzyme II (ACE2)^21^. Another study that analyzed COVID-19 allergic children and compared them to non-allergic children found no difference between the groups regarding the severity of the disease^22^.

When assessing lung tissue expression of TGF-β, there was no significant difference between the groups (Fig. 1 and Table 2). Patients of COVID-19 and H1N1 group have developed DAD with hyaline membranes in the alveoli, which have a strong tendency to organize in 2–4 weeks. Consequently, even if there is a lower lung tissue expression of TGF-β in this samples, the presence of some areas of recent fibrosis in patients with more than two weeks of mechanical ventilations indicate that TGF-β may be participating of the transition from acute to organizing DAD, suggesting remodeling induced by Th2 response. This lower lung tissue expression of TGF-β may also suggest that the proliferative Th2 phase has not been triggered in most patients.

Since a significant expression of IL-4 is observed in the COVID-19 group compared to H1N1 and CONTROL group (*p* = 0.003 and *p* = 0.05, borderline) and a lower expression of IL-13 and Sphingosine-1 in this group when compared with H1N1 (*p* = 0.007 and *p* = 0.05 respectively), it is suggested that IL-4 may be secreted not only by the Th2 pathway but before it. In some situations, such as extracellular infections, IL-4 is produced by mast cells induced by interleukin 33 (IL-33) and release of immunoglobulin E (IgE)^9^. These cells produce IL-4 independently of the signal transducer and transcription activator 6 (STAT 6), necessary for the differentiation of Th2 cells, which means that they can influence the differentiation of T cells in Th1 and Th2 type responses, being able to modulate the proliferation and production of cytokine in TCD8^+^ lymphocytes responses^23^. In this work, the IL-4 lung tissue expression was higher in most of the COVID-19 patients since the beginning of the aggravated disease despite the time from hospitalization to death and mechanical ventilation, suggesting that these mechanisms could be considered (Fig. 2). A study demonstrated a significant increase in mast cells in post-mortem biopsies alveolar septa of patients affected by COVID-19. It is suggested that its increased presence is strongly involved in the physiopathology of this disease^24^.

Recent SARS-CoV-2 studies report that the modulation of the Th1 response is remarkably reduced, given the low activation of TCD8^+^ cells, which appears to stimulate the secretion of Th2 cytokines, suppressing Th1/Th17-mediated inflammation^25–28^ . These findings support the study in question, considering the presence of the Th2 response, in addition to observing little neutrophil recruitment, which demonstrates that the origin of the response inflammatory effect of SARS-CoV-2 differs from that found in H1N1pdm09^29^. This tendency of Th1 response in H1N1pdm09 infection is observed in studies that demonstrated a significant serum dosage of IL-10, which would promote an increase in the number of Th1 cells, production of INF-γ, and a decrease in IL-4 levels^30,31^, besides, to increase of TCD8^+^ cells in the pulmonary profile in severe cases^32^.

Inflammatory cytokines have been the key mediators in the innate immune response and inflammatory reaction in both Middle East Syndrome Coronavirus (MERS-CoV) and SARS-CoV-2. Interestingly, patients with MERS-CoV have presented an immune response lacking on Th1 cells and directed towards a Th2 immune response, and studies have related the exacerbated Th2 response to more aggressive forms of its disease^33^.

Our study’s major limitation was the small number of samples analyzed. It is crucial to note out that the static information of autopsy data cannot reconstruct disease evolution. However, this analysis's strength is the comparison between two pandemic viruses responsible for lung injury but probably using distinct immune mechanisms^34^.

Considering the current pandemic scenario caused by SARS-CoV-2, numerous therapies have been proposed as an alternative to prevent the evolution of the disease. Researchers and health service providers strive to find the best therapeutic strategies, but so far, using off-label drugs has been the immediate alternative^35^. In these circumstances, the antiviral against virus RNA, Remdesivir, has been a potential therapy against SARS-CoV-2 without definitive results^36^. Heparin has also been used in the treatment of patients with COVID-19, and it has been actively used since it combats coagulopathies, which are most likely caused by the immune system decompensation when facing COVID-19^37^.

Since severe COVID-19 can lead to DAD, which has the potential of developing septal fibrosis, recovering patients may have an impairment of their lung functions, directly affecting their life quality. Considering that it is an irreversible condition, the use of monoclonal antibodies aimed at inhibiting Th2 cytokines could be used as a treatment for COVID-19.

Summarizing, although H1N1pdm09 activates the Th2 response, its pathogenesis seems to be strictly linked to the Th1/Th17 responses. In contrast, SARS-CoV-2 seems to promote lung damage through different mechanisms, such as the scarce participation Th1/Th17 response, and the higher participation of the Th2, when combined, might be inefficient for viral clearance. Thus, the understanding and management of the aggravated and ineffective immune response elicited by SARS-CoV-2 merit further clarification.

## Data Availability

All data generated or analyzed during this study are included in this published article.
